# Identification of a novel X-linked arginine-vasopressin receptor 2 mutation in nephrogenic diabetes insipidus: Case report and pedigree analysis

**DOI:** 10.1097/MD.0000000000017359

**Published:** 2019-10-04

**Authors:** Danxia Peng, Ying Dai, Xuan Xu

**Affiliations:** a Hunan Provincial People's Paediatric Medicine Center; b Key Laboratory for Regenerative Medicine of Ministry of Education, Jinan University, China.

**Keywords:** *AVPR2*, gene sequencing, mutation, nephrogenic diabetes insipidus, precision medicine

## Abstract

**Introduction:**

The clinical and genetic characteristics of nephrogenic diabetes insipidus (NDI) were described via assessing 2 cases of NDI patients from a Chinese family.

**Patient concerns:**

Two patients who manifest polyuria and polydipsia were admitted to hospital for definite diagnosis.

**Diagnosis:**

Water deprivation-vasopressin tests showed that the patients may possess renal-origin diabetes insipidus. All the levels of thyroid-stimulating hormone, luteinizing hormone, follicle stimulation hormone, adrenocorticotropic hormone, prolactin, and growth hormone in both patients were normal. These results were certified that both patients possess a nephropathy-type diabetes insipidus. B-mode ultrasonography and urinalysis test demonstrated that the patient's diabetes insipidus is unlikely to originate from renal organic disease. Remarkably, by nucleotide sequencing, we found a novel mutation c.414_418del in arginine-vasopressin receptor 2 (*AVPR2*) was related to the disease of NDI.

**Interventions:**

Two patients were treated with oral hydrochlorothiazide and indomethacin. In addition, low salt diet and potassium supplementation throughout the patients’ treatment.

**Outcomes:**

The clinical symptoms of 2 patients were significantly reduced after targeted therapy.

**Conclusion:**

A mutation in *AVPR2* was discovered to be associated with NID. It provides a new target for molecular diagnosis of NDI, enabling families to undergo genetic counseling and obtain prenatal diagnoses.

## 1 Introduction

Nephrogenic diabetes insipidus (NDI) is a human kidney disease affecting the urine-concentrating ability of the kidney. This stems from an inability to respond to the antidiuretic hormone, arginine vasopressin, resulting in a massive excretion of diluted urine. NDI patient manifest polyuria and polydipsia.^[1]^ The symptoms of NDI range in their severity between individuals, owing to the heterogeneity of the mutations responsible for the disorder.^[2]^ Approximately 90% of patients with an X-linked form of NDI (OMIM 304800), which affect male patients, are unable to concentrate their urine in response to the antidiuretic hormone, arginine-vasopressin (AVP). This is caused by mutations in the AVP receptor 2 gene (*AVPR2*), while the remaining 10% of patients have an autosomal form of NDI (OMIM 125800), caused by mutations in the aquaporin 2 gene (*AQP2*).^[3–8]^

According to other scientific findings, over 200 disease-causing *AVPR2* mutations had been published, comprising missense, nonsense, small insertions and deletions, large deletions, and complex rearrangements.^[9]^ Those mutations have been identified in more than 300 congenital NDI families, about 56% of which were missense mutations.^[10]^ Molecular diagnosis is the best method for the diagnosis of NDI and studying its genetic mechanisms. Due to the heterogeneity of NDI, we performed exon capture sequencing using next-generation sequencing (NGS), to identify the genetic features of 2 brothers with NDI clinical characteristics. We discovered a novel mutation in *AVPR2*, and performed the Sanger sequencing for verification. Identification of the patient's clinical characteristics combined with the pedigree verification leads us to believe that we have found 2 patients who conform to X-linked NDI. To the best of our knowledge, this was the first report of the mutation, both nationally and internationally, presenting great significance for the epidemiological study of NDI in China. Owing to the progressive development of techniques for molecular diagnosis, the disease is now diagnosed in most patients at an early age, which is beneficial to the treatment of congenital NDI.^[11,12]^ Meanwhile, our findings will further supplement the databases of genetic diseases in China, contribute to the diagnosis of diseases, and promote the development of precision medicine, such as preimplantation genetic screening, preimplantation genetic diagnosis, prenatal screening, as well as prenatal diagnosis.

## 2 Materials and methods

### 2.1 Collection of medical history and related auxiliary examination

This study enrolled 2 patients; an 18-year-old male (Patient 1) and his 9-year-old brother (Patient 2), whose were suspected to be afflicted with NDI, from the 7th Pediatric Department and Outpatient Department of Pediatrics of Hunan Provincial People's Hospital, in February 2017. The medical history, physical examination data, as well as related auxiliary inspection data, were collected after obtaining informed consent from the parents and approval from the Ethics Committee of the hospital and Hunan Normal University. Written informed consent for molecular analysis was obtained from the patient or in the case of children, from their parent or legal guardian. The study was approved by the Clinical Research Ethics Committee, People's Hospital of Hunan province and Hunan Normal University (NM: 2017–16).

### 2.2 Evaluation of gene virulence

#### 2.2.1 Sample collection

On the provision of informed consent, 4-mL whole blood samples were collected from the patient, his brother, and his parents, using an EDTA anticoagulant; thereafter, genomic DNA of the patient was extracted using the BloodGen Midi Kit (Beijing ComWin Biotech Co, Ltd, China), as per manufacturer's instructions and as previously described.^[13]^

#### 2.2.2 Target sequence capture and DNA sequencing

For whole-exome capture of the target genes, the NimbleGen capture probe (Roche, Switzerland) was prepared for genomic exon regions relevant to >4000 genetic diseases based on information from existing literature and the OMIM and Orphanet databases (Table 1).

**Table 1:**
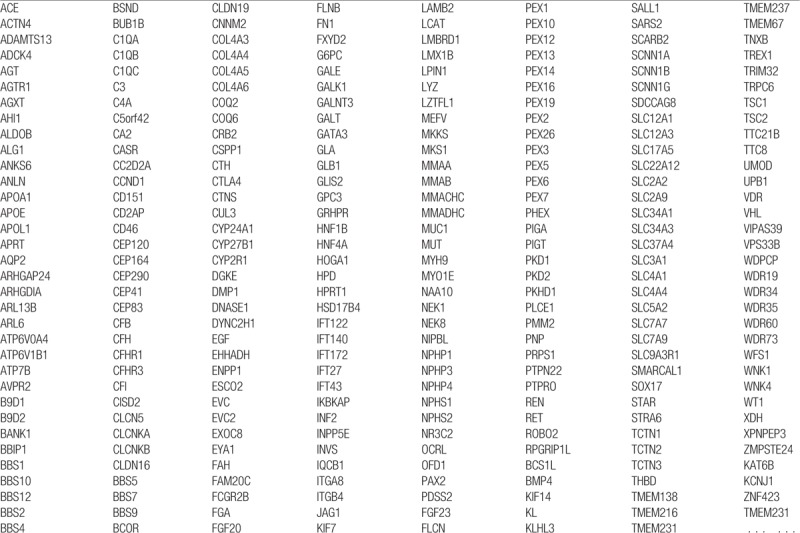
Part of the gene panel of this study.

##### 2.2.2.1 Library preparation

Genome fragmentation: Using Cavoris auxiliary reagent for Illumina, genes were fragmented into approximately 200-bp fragments.

Free-end filling-in and repair: This was performed for the DNA fragments using the Klenow fragment, T4 DNA polymerase, and T4PNK.

3′-terminal adenosine addition: In the polymerase system, an A base was added to the 3′-terminal of the repaired products obtained in the preceding step to prepare them for connection in the subsequent step.

Connector addition: The T4DNA ligase reaction system was configured, while adapter and supplementary “A” products were connected after reaction at the appropriate temperature for a certain period in the ThermoMixer.

Amplification: The ligation products were subjected to 4 to 6 rounds of ligation-mediated polymerase chain reaction (LM-PCR) amplification.

Hybridization: Library and probes were mixed in a hybridization system at 65°C for 60 to 68 hours.

Bead washing and DNA elution: Streptomycin beads were incubated with the hybridized samples, following which elution was performed.

Eluted product amplification: Eluted products were amplified by 10 rounds of LM-PCR.

##### 2.2.2.2 Illumina sequencing

(1) Sequencing: The operational process of sequencing was standardized by using the Illumina hiseq2500 platform.
(2) Raw image data obtained by sequencing were analyzed using the Illumina official base call analysis software, BclToFastq.

##### 2.2.2.3 Data analysis

Analysis of base data:

(1) Optimization of raw data production: connector contamination and low-quality data were excised.
(2) Comparison: Comparative statistics were applied to the data and reference sequence (Burrows–Wheeler Alignment software was used); the hg19 genome was used as reference.
(3) SNP detection and annotation were performed using the Samtools software.
(4) Indel detection and annotation were performed using the Pindel software.
(5) False-positive mutation filter: to obtain high quality and reliable mutations, the detected SNPs and indels were filtered and screened based on sequencing depth and mutation quality.
(6) Mutation annotation: SNPs and indels were analyzed to obtain the impact of amino-acid changes, shear effects, UTR, and intron mutations, according to gene location.
(7) Prediction of effects of screened mutations on protein function: The impact of screened mutations on protein was predicted by SIFT, the homology alignment algorithm, and protein structure conservation.
(8) The splicing hazard was predicted for mutations near splice sites.

In-depth data analysis was conducted to associate mutations in related genes with their genetic patterns and clinical symptoms matching those in Patient 1.

#### 2.2.3 Verification of mutations using Sanger sequencing

Primers were designed according to the sequences of sites, validated by *AVPR2*. Amplification was performed using PCR, and sequencing was conducted with the ABI 3730XL sequencing device. The original PCR primers were used for sequencing. Genetic sequence analysis and alignment were performed using DNASTAR software, and the messenger RNA alignment template was NM_003560. Samples from Patient 1, Patient 2, and their parents were validated using first-generation sequencing.

## 3 Results

### 3.1 Clinical data

Two patients were admitted to hospital due to their manifest polyuria and polydipsia. Patient 1 had an input water quantity of 4770 mL/d, and an output urine volume of 5200 mL/d, which is within diabetes insipidus diagnostic criteria. At the same time, Patient 2 had an input water quantity of 5850 mL/d, and an output urine volume of 6750 mL/d, which also met the diagnostic criteria of diabetes insipidus. They were born without asphyxia at birth and their mother had no special medical history of pregnancy.

### 3.2 Family history

**Figure 1:**
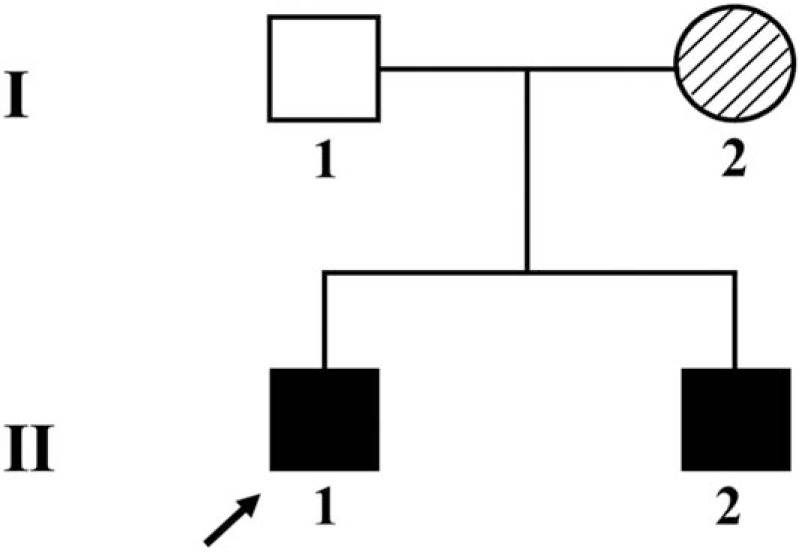
A simply pedigree map of the patients’ family.

The parents of the patients were healthy without consanguineous marriage. There was no consultable history of special genetic diseases within this family. Both patients presented similar clinical characteristics (Fig. 1).

### 3.3 Water deprivation-vasopressin test

In order to confirm whether the patients possessed psychogenic polydipsia or diabetes insipidus, both patients underwent a water deprivation-vasopressin test. The results of Patient 1 are shown in Table 2. The results indicated that after water deprivation, the patient displayed a decrease in weight and blood pressure, while serum Na^+^ levels and blood osmotic pressure showed an increase. The patient showed little change in urine output, and urine osmolality was still maintained at a very low level. Based on these characteristics, we concluded that the patient could be distinguished from psychogenic polydipsia and the normal population. Furthermore, Patient 1 showed a stable level of urine output, urine gravity, urine osmotic pressure, and blood osmolality after injected pituitrin. It was, therefore, demonstrated that the patient had no response to pituitary vasopressin, so was considered to possess renal-origin diabetes insipidus. The results for Patient 2 were found to be similar to those of Patient 1 (Table 3). In conclusion, both patients presented with diabetes insipidus, rather than psychogenic polydipsia. Furthermore, the results of both patients’ vasopressin tests, showed that the patients may possess renal-origin diabetes insipidus.

**Table 2:**
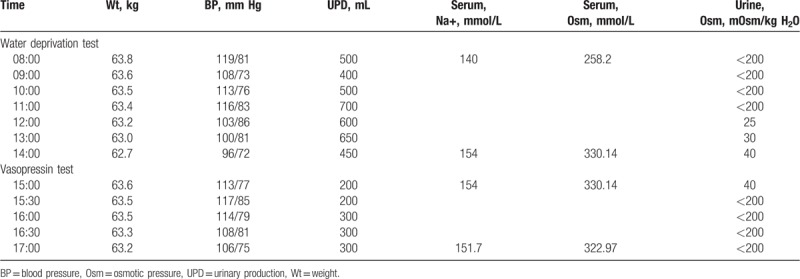
Results of water deprivation – vasopressin test of Patient 1.

**Table 3:**
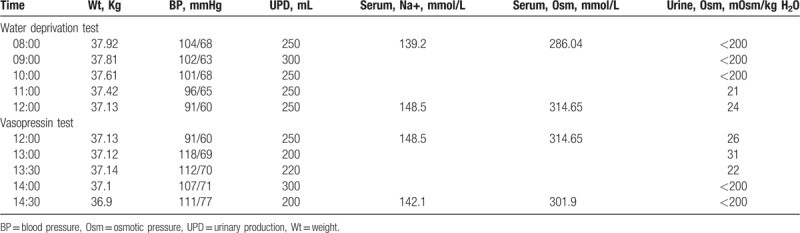
Results of water deprivation – vasopressin test of Patient 2.

### 3.4 Magnetic resonance imaging

**Figure 2:**
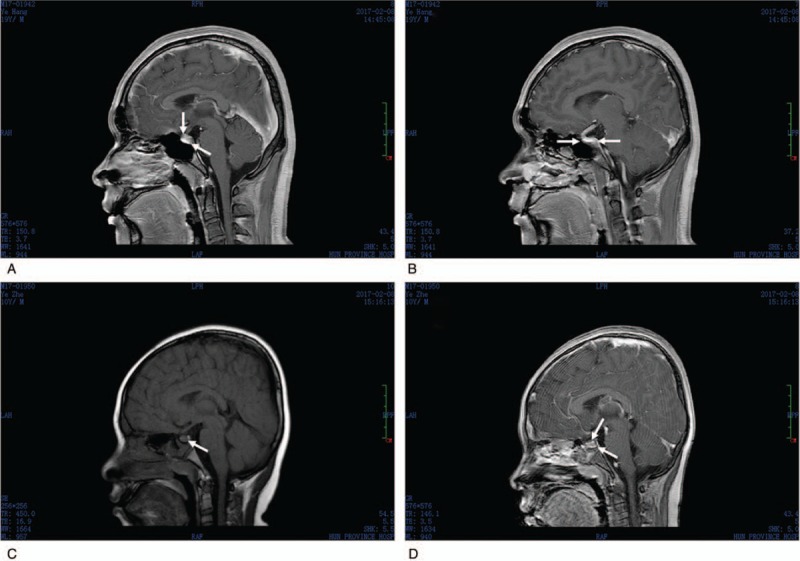
The MRI results from the patients. Patient 1 with cysts at sinusoid (A, B); Patient 2 showed a large amount of fluid stored within the sinus cavity, resulting in sinusitis (C, D). MRI = magnetic resonance imaging.

In order to confirm whether the patients were suffering from a central nervous-origin diabetes insipidus, we performed magnetic resonance imaging (MRI). The results showed that Patient 1 displayed a microadenoma at the pituitary body (Fig. 2A and B), but that his pituitary signal was normal. The results for Patient 2 showed a large quantity of fluid stored within the sinus cavity, confirming the presence of a nasosinusitis (Fig. 2C and D), while his pituitary signal was also normal. As the MRI results showed that both patients displayed a normal pituitary signal, it provided powerful evidence that their clinical features do not originate from a central nervous problem. However, it is difficult to confirm whether pituitary adenoma (Patient 1) and nasosinusitis (Patient 2), have no effect on the posterior pituitary or vasopressin storage and secretion.

### 3.5 Pituitary hormone detection

In order to confirm whether both patients’ posterior pituitary glands display normal levels of secretion, we tested their pituitary hormone levels. The results showed that the levels of thyroid-stimulating hormone, luteinizing hormone, follicle stimulation hormone, adrenocorticotropic hormone, prolactin, and growth hormone in both patients were all normal (Table 4). This confirms that both patients possess a nephropathy-type diabetes insipidus.

**Table 4:**
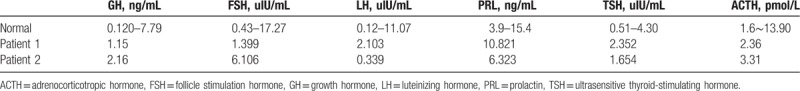
Results of pituitary hormone detection.

### 3.6 B-mode ultrasonography and urinalysis test

**Figure 3:**
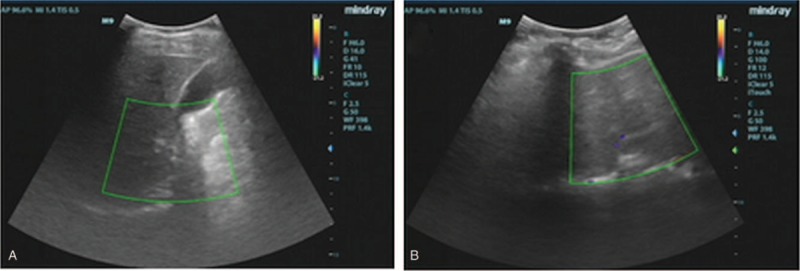
The type-B ultrasound results from the patients. There were no obvious abnormalities in the kidneys of Patient 1 (A) or Patient 2 (B).

In order to identify whether the patients’ diabetes insipidus originated from a renal disease or a genetic defect, both patients were subjected to a urinary B-mode ultrasonography and urinalysis test. The B-mode ultrasonography results showed that there were no obvious abnormalities in the kidneys of either patient (Fig. 3), while the urinalysis test results showed that their kidney function was normal (Tables 5 and 6). This demonstrated that the patient's diabetes insipidus is unlikely to originate from renal organic disease.

**Table 5:**
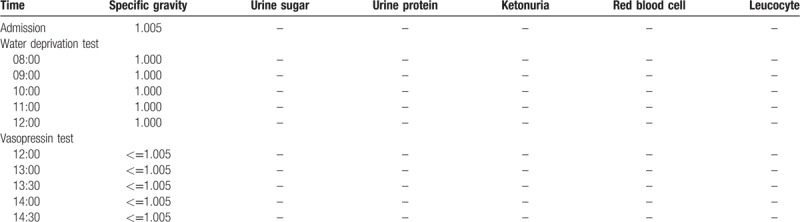
Results of urinalysis tests of Patient 1.

**Table 6:**
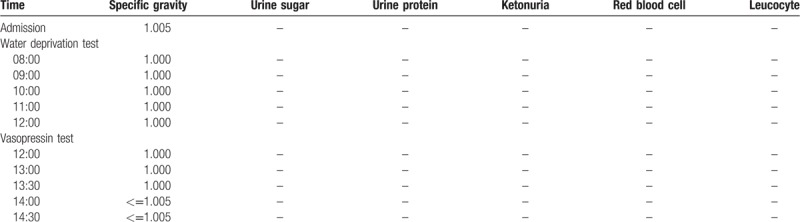
Results of urinalysis tests of Patient 2.

### 3.7 Conclusion of examination upon admission

It was noted from the clinical data that both patients suffered from polyuria and polydipsia, in line with the clinical diagnostic criteria of NDI. In addition, the patients presented with the clinical characteristics of hypophyseal adenoma and normal pituitary hormone levels. It is therefore difficult to confirm the patients’ phenotype, according to classical clinical knowledge, and the differential diagnosis of this case required further molecular genetic analysis.

## 4 Genetic detection

**Figure 4:**
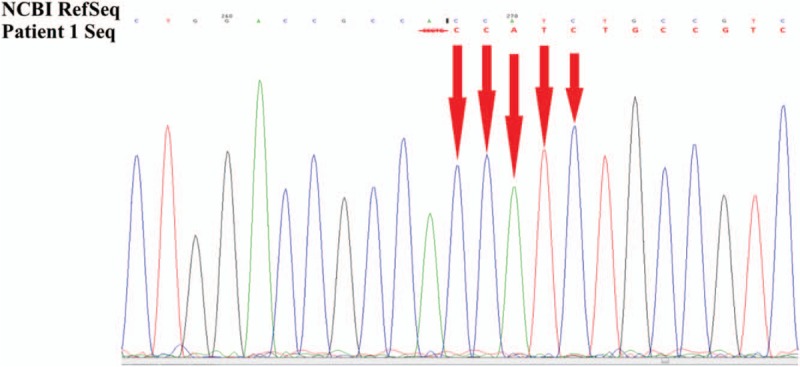
The genetic detection results of Patient 1. A hemizygous mutation c.414_418del was found in *AVPR2* in the Patient 1. AVPR2 = arginine-vasopressin receptor 2.

**Figure 5:**
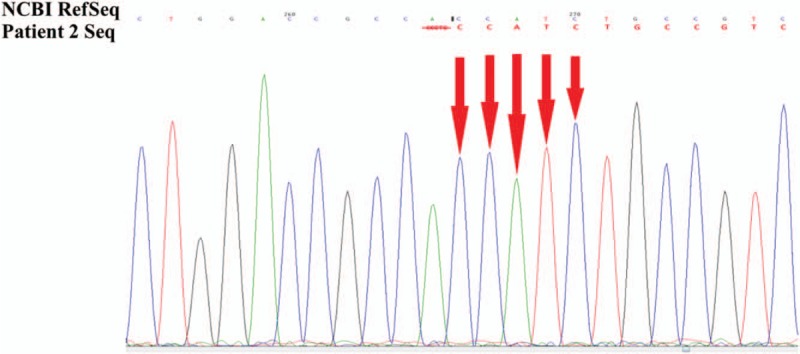
The genetic detection results of Patient 2. A hemizygous mutation c.414_418del was found in *AVPR2* in the Patient 2. AVPR2 = arginine-vasopressin receptor 2.

**Figure 6:**
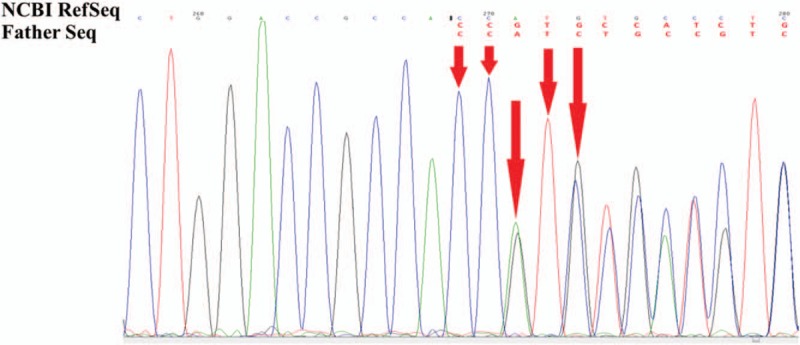
The genetic detection results of the patients’ father. The patients’ father displayed the wild-type gene at the locus.

**Figure 7:**
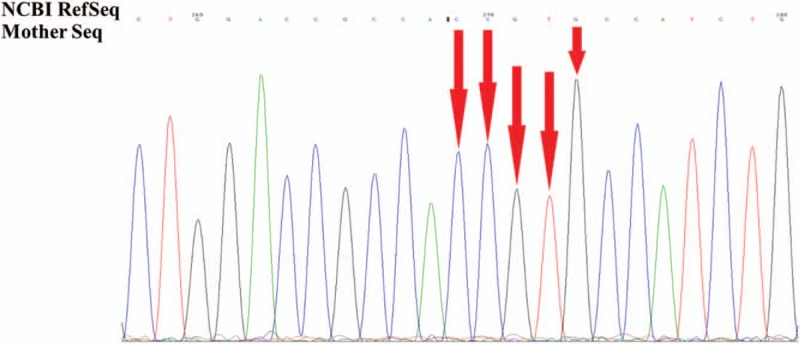
The genetic detection results of the patients’ mother. A heterozygous mutation c.414_418del was found in *AVPR2* of the patients’ mother. AVPR2 = arginine-vasopressin receptor 2.

**Figure 8:**
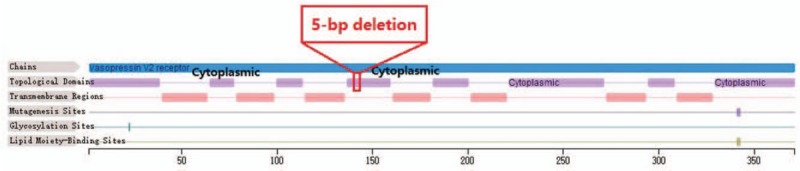
The location of the novel mutation at *AVPR2*. AVPR2 = arginine-vasopressin receptor 2.

Suspicious mutant sites were found to be present in *AVPR2* in the pediatric patient, by using high-throughput, exon-capture sequencing, bioinformatic analysis, and clinical database analysis. Validation using first-generation sequencing and pedigree verification were also performed. The results were as follows; A novel mutation c.414_418del was found in *AVPR2* in Patient 1 (Fig. 4). His younger brother possessed the same genotype (Fig. 5), while his mother possessed a hemizygous mutation at this locus (Fig. 6), and his father was wild-type (Fig. 7). This novel mutation is a type of 5-bp deletion within *AVPR2*, located in the coding region for the second cytoplasmically localized domain (Fig. 8). The deletion results in a frameshift mutation, and may contribute to nonfunctional protein being expressed, leading to abnormal cell function. Genetic detection and pedigree validation revealing that Patient 1, Patient 2, and their mother were mutant types, while their father was wild-type, was consistent with the X-linked inheritance pattern of NDI (Fig. 1). The distribution frequency of this mutation was not found in the dbSNP database, Hapmap, 1000 Genomes database, or ExAC. This mutation was, therefore, a novel mutation that had not been previously reported in the relevant literature.

**Figure 9:**
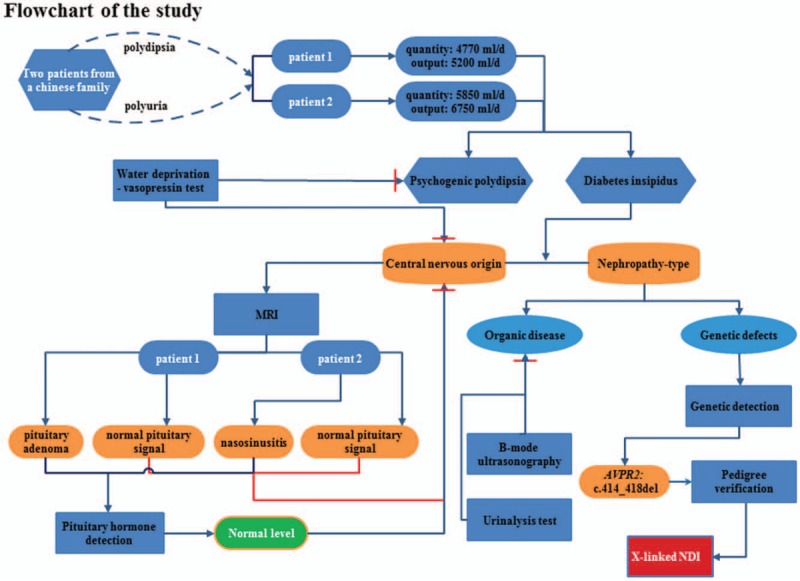
A flowchart of the study.

In summary, Patient 1 and Patient 2 may suffer from NDI, combined with some of the clinical characteristics of hypophyseal adenoma and nasosinusitis, respectively. Molecular genetic analysis found the presence of a mutation in *AVPR2*, but the mechanism of additional clinical characteristics needs to be studied further in more cases. A flowchart of the study is shown in Figure 9.

## 5 Discussion

A number of families of patients suffering from the X-linked form of NDI, have been studied genetically. The gene responsible for congenital NDI has been mapped to the long arm of the X chromosome, near the Xq28 marker, where it colocalizes with *AVPR2* (chrX: 153,170,428-153,172,620).^[14]^ The whole genomic locus of *AVPR2* is 2,193 bp in length, consisting of 3 exons, within which mutations have been found to be responsible for congenital NDI.^[15–20]^

The gene encoding the vasopressin type-2 receptor, also known as the V2 receptor, belongs to the 7-transmembrane-domain G protein-coupled receptor superfamily, and couples to G proteins to stimulate adenylate cyclase activity.^[14]^ The subfamily that includes the V2 receptor as well as the V1a and V1b vasopressin receptors, the oxytocin receptor, and isotocin and mesotocin receptors in nonmammals, is well conserved, though several members carry out signaling via other G proteins. The V2 receptor is expressed in the kidney tubule, mainly in the distal convoluted tubule and collecting ducts, where its primary function is to respond to the pituitary hormone, arginine vasopressin, by stimulating mechanisms that concentrate urine and maintain water homeostasis in the organism. Loss-of-function of this gene results in NDI disease. X-linked congenital NDI is caused by the loss of, or decreased function of, *AVPR2*, and large deletions that lead to the complete loss of *AVPR2* have been reported comprehensively. Furthermore, mutations to *AQP2*, *ARHGAP4*, and *L1CAM* have also been reported to result in NDI.^[21–24]^ However, previous studies have indicated that variations in different genes lead to distinct clinical phenotypes.^[25]^ Therefore, clinical differential diagnosis and the detection of variation are very necessary for the diagnosis of NDI.

In this study, we have identified a novel type of 5-bp deletion in *AVPR2* in 2 Chinese patients with NDI from the same family who display similar clinical characterization and symptoms. The parents of the patients were healthy and there was no known history of special genetic diseases in this family. The patients manifested polyuria and polydipsia, and met the criteria for a diagnosis of diabetes insipidus. The results of a water deprivation – vasopressin test excluded the possibility of psychogenic polydipsia. The MRI results showed that both patients displayed a normal pituitary signal, and provided powerful evidence to demonstrate that their clinical features did not originate from central nervous problems. The water deprivation – vasopressin test results and the normal pituitary hormone levels of 2 patients verified these conclusions. The novel mutation of the patients is a 5-bp deletion in *AVPR2*, located in the second-transmembrane-domain. The deletion results in a frameshift mutation, and may contribute to the loss of protein function, leading to abnormal cell function. Finally, we affirmed that the children suffered from X-linked NDI, through performing NGS and family lineage verification.

We have demonstrated that a mutation in *AVPR2* is the cause of NDI in the great majority of congenital NDI families.^[26]^ In families in which an *AVPR2* mutation has been identified, NDI is caused by the mutation in about 90% of cases. There remain a few NDI families for which a mutation has not been identified. Based on recent improvements in NGS techniques, mass DNA sequencing (including full exon sequencing or whole genome sequencing) is progressively being used in the antidiastole of NDI. Currently, genetic testing is used for precise diagnosis, such as; Sanger sequencing, amplification refractory mutation system-polymerase chain reaction, and Droplet Digital PCR are all used to identify the sequences of certain genes. However, the number of genes is very high, and the above methods are costly, with lengthy run times. This makes them very difficult to use in clinical practice, especially for diagnosing diseases with similar characteristics. NGS could provide high-throughput sequencing of multiple genes, including all genes associated with a given disease, thus reducing costs and run times. Therefore, NGS is suitable for screening genes associated with hereditary diseases showing similar clinical characteristics.^[27]^

In the present study, we used a chip capturing high-throughput sequencing method for whole-exome sequencing of monogenic pulmonary disease-related genes, and successfully identified a virulence gene site in 1 pedigree. To our knowledge, this novel mutation is the first report about *AVPR2*, which has a certain value for the epidemiological investigation of NDI in China. In addition, the identification of this gene site could further complement the Hereditary Disease Library of China. Currently, dozens of monogenic diseases could be screened in China, which would effectively reduce the risk of birth defects. In addition, precise classification and diagnosis provides the foundation for disease assessment, and a basis for prenatal screening, prenatal diagnosis, and genetic counseling. We believe that with the accumulation of cases and genetic data in China, birth defects, including NDI, could be effectively avoided in the future.

## Author contributions

**Data curation:** Xuan Xu, Ying Dai, Danxia Peng.

**Formal analysis:** Xuan Xu, Danxia Peng.

**Funding acquisition:** Xuan Xu.

**Methodology:** Xuan Xu.

**Project administration:** Xuan Xu, Ying Dai.

**Software:** Ying Dai.

**Writing – original draft:** Xuan Xu, Ying Dai.

**Writing – review & editing:** Ying Dai.

Xuan Xu orcid: 0000-0001-5543-2650.
